# Deficiency of biodegradable plastic-degrading enzyme production in a gene-deletion mutant of phyllosphere yeast, *Pseudozyma antarctica* defective in mannosylerythritol lipid biosynthesis

**DOI:** 10.1186/s13568-019-0825-2

**Published:** 2019-07-06

**Authors:** Azusa Saika, Hideaki Koike, Tohru Yarimizu, Takashi Watanabe, Hiroko Kitamoto, Tomotake Morita

**Affiliations:** 1 Research Institute for Sustainable Chemistry, National Institute of Advanced Industrial Science and Technology (AIST), Tsukuba Central 5-2, 1-1-1 Higashi, Tsukuba, Ibaraki 305-8565 Japan; 2 Bioproduction Research Institute, National Institute of Advanced Industrial Science and Technology (AIST), Tsukuba Central 6-9, 1-1-1 Higashi, Tsukuba, Ibaraki 305-8566 Japan; 3 Institute for Agro-Environmental Sciences, National Agricultural Food Research Organization (NARO), 3-1-3 Kannondai, Tsukuba, Ibaraki 305-8604 Japan; 4 Present Address: Gunma Industrial Technology Center, 884-1 Kamesato, Maebashi, Gunma 379-2147 Japan

**Keywords:** *Pseudozyma antarctica*, Esterase, Biodegradable plastic, Mannosylerythritol lipid, Glycosyltransferase, Gene deletion

## Abstract

The basidiomycetous yeast *Pseudozyma antarctica* (currently designated *Moesziomyces antarcticus*) produces extracellular enzymes and glycolipids, including mannosylerythritol lipids (MELs), which are biosurfactants. Strain GB-4(0) of this species was previously isolated from rice husks and produces biodegradable plastic-degrading enzyme (*Pseudozyma antarctica* esterase; PaE). In this study, we generated a MEL biosynthesis-deficient strain (∆Pa*EMT1*) by deleting the gene Pa*EMT1*, which is essential to MEL biosynthesis in strain GB-4(0). The resulting ∆Pa*EMT1* strain showed deficient PaE activity, and the corresponding signal was hardly detected in its culture supernatant through western blotting analysis using rabbit anti-PaE serum. On the other hand, the relative expression of the gene Pa*CLE1*, encoding PaE, was identical between GB-4(0) and ∆Pa*EMT1* based on quantitative real-time PCR. When strain ∆Pa*EMT1* was grown in culture media supplemented with various surfactants, i.e., Tween20, BRIJ35 and TritonX-100, and MELs, PaE activity and secretion recovered. We also attempted to detect intracellular PaE using cell-free extract, but observed no signal in the soluble or insoluble fractions of ∆Pa*EMT1*. This result suggested that the Pa*CLE1* gene was not translated to PaE, or that expressed PaE was degraded immediately in ∆Pa*EMT1*. Based on these results, MEL biosynthesis is an important contributor to PaE production.

**Electronic supplementary material:**

The online version of this article (10.1186/s13568-019-0825-2) contains supplementary material, which is available to authorized users.

## Introduction

*Pseudozyma antarctica* (currently designated *Moesziomyces antarcticus*) has the ability to produce some materials, including enzymes and glycolipids (Boekhout and Fell 1998). In recent years, we have focused on an esterase (*Pseudozyma antarctica* esterase; PaE) that degrades biodegradable plastics (BP), including poly(butylene succinate) (PBS), poly(butylene succinate-*co*-adipate) (PBSA), poly(ε-caprolactone) (PCL), and poly(lactide) (PLA) (Kitamoto et al. 2011; Shinozaki et al. 2013). *P. antarctica* strains isolated from rice husks secrete relatively high amounts of PaE into the culture supernatant, and PaE production is strongly enhanced when these strains are cultivated with xylose (Watanabe et al. 2014). PBSA, PBS and commercially available BP mulch films submerged in the culture supernatant of *P. antarctica* are rapidly degraded (Watanabe et al. 2014). To accelerate PaE utilization, we constructed a recombinant strain that is able to produce large amounts of PaE (13.4-fold higher amount than that of the wild-type strain) (Watanabe et al. 2016). Additionally, a targeted gene manipulation method has been developed recently (Yarimizu et al. 2017) that allows more efficient modification of the PaE production of this strain.

*Pseudozyma antarctica* is well known to produce glycolipid-type biosurfactants known as mannosylerythritol lipids (MELs), which are composed of mannose, erythritol and fatty acids. MELs show excellent interfacial properties, as well as moisturizing activity toward cultured human skin cells; therefore, they have been used commercially in skincare products and cosmetics (Yamamoto et al. 2012). MEL production by *P. antarctica* was first reported in strain T-34, isolated from the exudate of a tree on Mt. Tsukuba in Japan (Kitamoto et al. 1990). The genomic sequence of *P. antarctica* T-34 has been reported, and five genes (Pa*EMT1*, Pa*MAC1*, Pa*MAC2*, Pa*MAT1* and Pa*MMF1*) involved in MEL biosynthesis have been identified (Morita et al. 2013a).

Recently, we found that the BP film degradation activity of PaE was inhibited by MELs through in vitro analysis using surface plasmon resonance (Fukuoka et al. 2016). The hydrophobic lipid domain of MEL interacted with the BP film, and the hydrophilic sugar domains of MEL were regularly oriented on the MEL-coated film surface. The hydrophobic portion of PaE attaches to hydrophobic substrates such as BPs, as interaction between the enzyme and substrate is crucial for the degradation activity (Shinozaki et al. 2013). The negative effects of synthetic surfactants on production of esterases by yeasts have been studied in several previous papers. Namely, production of lipases, a subclass of esterases, by the yeasts *Candida viswanathii* and *Yarrowia lipolytica* was suppressed with the addition of either Tween80 or TritonX-100 (Almeida et al. 2013; Dominguez et al. 2003). Thus, we speculated that MELs biosynthesized by the host strain would also have negative effects on PaE production, and that deletion of MEL biosynthesis genes may eliminate these negative effects.

To remove the ability to MEL biosynthesis, we focused on the gene Pa*EMT1*, encoding glycosyltransferase, because this gene is essential for MEL biosynthesis. A glycosyltransferase-deletion strain of the smut fungus *Ustilago maydis* and *P. antarctica* strain T-34 were completely defective in MEL biosynthesis (Hewald et al. 2005; Morita et al. 2010). In this study, we constructed a MEL biosynthesis-deficient strain through deletion of Pa*EMT1* in GB-4(0). Unexpectedly, the Pa*EMT1*-deletion strain was defective in the production of both MEL and PaE. The gene Pa*CLE1*, encoding PaE, was expressed normally, regardless of MEL biosynthesis. The effect of MEL on PaE production was investigated in the Pa*EMT1*-deletion strain with and without exogenous surfactants, and the deficiency of enzyme production was recovered with the addition of MEL and other synthetic surfactants to the culture medium. Therefore, we speculated that either the Pa*CLE1* gene was not translated to PaE or expressed PaE was degraded immediately in strain ∆Pa*EMT1*. This is the first report focused on their production between extracellular enzyme and glycolipid using a MEL biosynthesis deficient strain.

## Materials and methods

### Strains and plasmid

The strains and plasmids used in this study are listed in Table 1. *Pseudozyma antarctica* strain GB-4(0) (Kitamoto et al. 2011; Accession No. MAFF 306999) and Pa*EMT1*-deletion strain Pa*EMT1*∆::NAT (∆Pa*EMT1*) of GB-4(0) were employed as the host strains for PaE production. The ΔPa*EMT1* strain was generated as described below. Pa*EMT1* from the *P. antarctica* T-34 (Accession No. DF196785) expression plasmid pUXV1_neo-PaEMT1 was using for complementation of ΔPa*EMT1* (Morita et al. 2013b).

**Table 1:** Strains, plasmids and primers used in this study

Strains, plasmids, primers	Description	Reference/source
Strains		
*P. antarctica* GB-4(0)	Wild type strain isolated from rice husks and stocked in our laboratory	Kitamoto et al. (2011)
*P. antarctica* GB-4(0) ∆*PaEMT1*	Pa*EMT1* deleted strain	In this study
Plasmids		
pAG25	Dominant marker gene deletion cassette: pAgTEF1-natMX-tAgTEF1, clonNAT and ampicillin resistance	Goldstein and McCusker (1999)
pUXV1_neo	Expression plasmid, glyceraldehyde-3P-dehydrogenase (gap) promoter, geneticin (G418) and ampicillin resistance	Watanabe et al. (2016)
pUXV1_neo-PaEMT1	Inserted Pa*EMT1* from *P. antarctica* T-34 into pUVX1_neo	Morita et al. (2013b)
Primers		
PaEMT1_up_F1	GAAGAGGCAATGGTGTTGCT	In this study
PaEMT1_up_R1	CGGCGGGGACAAGGCAAGCTATTTGCTTGAGCGATATGCTTTTG	In this study
NAT_F1	AGCATATCGCTCAAGCAAATAGCTTGCCTTGTCCCCGCCGGGTCAC	In this study
NAT_R1	GTTTAATCAGGACAAGGCGTTCGACACTGGATGGCGGCGTTAGTATCG	In this study
PaEMT1_down_F1	ACGCCGCCATCCAGTGTCGAACGCCTTGTCCTGATTAAACTGCTG	In this study
PaEMT1_down_R1	CAATGTCATCCACAGCACTC	In this study
PaEMT1_inner_F1	CTGCATCGATTGATCCATTG	In this study
PaEMT1_inner_R1	GTCGAACCACTGCGACAGGA	In this study
PaEMT1_up_F2	ATTGCTGACCATCATGGTGC	In this study
NATfragment_R1	CACGTCAAGACTGTCAAGGA	In this study
PaCLE1_qPCR_F	TTCCGGACCATGAACACCCG	In this study
PaCLE1_qPCR_R	AGGAGGAAGCACGTGTTGGG	In this study
Actin_qPCR_F	CAGTCGTCTGCGCTCGAGAA	In this study
Actin_qPCR_R	CGGATGTCCAGGTCGCACTT	In this study

### Construction of the MEL biosynthesis-deficient strain

To obtain a strain that does not produce MEL, the gene Pa*EMT1* was deleted through homologous recombination in *P. antarctica* GB-4(0). The two gene fragments upstream (1.1-kb) and downstream (1.0-kb) of Pa*EMT1* were amplified via PCR with the primer set (PaEMT1_up_F1, R1 and PaEMT1_down_F1, R1) listed in Table 1, and with the genomic DNA of strain GB-4(0) used as a template. The natMX4 cassette with the nourseothricin acetyltransferase gene (*nat1*) controlled by *Ashbya gossypii* TEF promoter and terminator (1.2-kb) was amplified through PCR with the primer set (NAT_F1, R1) listed in Table 1, using plasmid pAG25 (Goldstein and McCusker 1999) as a template. The gene fragment natMX4, flanked by the sequences of the Pa*EMT1* locus, was amplified by overlap PCR using three fragments that share 40 base-pair end-terminal homology as templates, yielding fragment Pa*EMT1*::natMX4 (Additional file 1: Figure S1A), checked using gel electrophoresis, and purified with the MagExtractor-PCR & Gel Clean-up kit (TOYOBO Co., Ltd., Osaka, Japan). Approximately 10 μg of this DNA fragment was introduced into strain GB-4(0) using the lithium acetate method (Yarimizu et al. 2017). The transformed cells were grown on YPD medium agar plates containing 100 μg/mL nourseothricin (clonNAT; Jena Bioscience, Jena, Germany), and Pa*EMT1* deletion was confirmed using colony PCR.

### PaE production

Seed cultures of the strains were grown in 2 mL YM medium (3 g/L yeast extract, 3 g/L malt extract, 5 g/L peptone and 10 g/L glucose) in test tubes at 30 °C for 3 days with shaking at 250 strokes/min (reciprocal). Then, 300 μL of seed culture was inoculated into a 300-mL flask with 30 mL *modified*(*m*)-3×FMM (fungal minimum medium; 3 g/L yeast extract, 2 g/L NaNO_3_, 0.6 g/L KH_2_PO_4_ and 0.6 g/L MgSO_4_·7H_2_O) containing 8 (w/v)% xylose that modified from previous report (Watanabe et al. 2014). The strains were cultivated at 30 °C for 4 days with 200 strokes/min shaking (rotary). After cultivation, the culture was centrifuged at 20,400*g*, and its supernatant was used for the PaE activity assay. To evaluate the functional complementation of ΔPa*EMT1*, Pa*EMT1* was introduced using a plasmid that carries a bacterial neomycin resistance gene (*neo*), namely pUXV1_neo-PaEMT1, via the lithium acetate method, and allowing selection on YM medium containing 500 μg/mL geneticin (G418). 200 μg/mL G418 was added to the culture medium to maintain the plasmid. Cell growth was determined by measuring the optical density of samples at 600 nm (OD_600_). All cultivations were performed in triplicate.

### PaE detection through SDS-PAGE and western blotting

PaE production in the culture supernatant and cell-free extract were analyzed through sodium dodecyl sulfate polyacrylamide gel electrophoresis (SDS-PAGE) according to the method of Laemmli (1970), using a 14% polyacrylamide slab gel (culture supernatant) or Any kD Mini-Protean TGX gel (cell-free extract; Bio-Rad, Richmond, CA, USA). Proteins were visualized with Coomassie brilliant blue (CBB) staining using Quick-CBB (culture supernatant; FUJIFILM Wako Pure Chemical Corporation, Osaka, Japan) or SimplyBlue™ SafeStain (cell-free extract; Invitrogen, Carlsbad, CA, USA) kits. The proteins separated through SDS-PAGE were transferred to PVDF membranes using Trans-Blot SD Semi-Dry Transfer Cell (Bio-Rad) at 60 mA for 60 min. Detection of PaE through western blot analysis was performed using rabbit anti-PaE serum (1/3.5 × 10^4^) (Kitamoto et al. 2011) and HRP-linked donkey anti-rabbit polyclonal antibody (GE Healthcare, Little Chalfont, UK) as the primary and secondary antibodies, respectively. The PaE-antibody conjugate was detected with FluorChem and AlphaEase FC Software (Alpha Innotech, San Jose, CA, USA) using ECL western blotting detection reagent (GE Healthcare). Purified PaE was used as a control, as described previously (Suzuki et al. 2013).

### Preparation of cell-free extract

Preparation of the cell-free extract is summarized in Additional file 1: Figure S2. To prepare cell-free extract, cells were harvested from 1 mL culture broth through centrifugation at 9100*g* for 2 min. The cells were resuspended in 0.5 mL 20 mM Tris–HCl (pH 6.8) containing 1 mM PMSF, and then disrupted with two 5-mm and twenty 2-mm zirconia beads at 3200 rpm for 5 min (three times) using the Beads Crusher μT-12 (TAITEC, Saitama, Japan). After disruption, 0.5 mL 20 mM Tris–HCl (pH 6.8) wad added, and 0.8 mL of sample was transferred to a 1.5-mL tube. The cell debris was removed through centrifugation at 9100*g* for 2 min and re-suspended in 0.8 mL 20 mM Tris–HCl (pH 6.8) as the insoluble fraction. TritonX-100 was added to the insoluble fraction (final concentration: 0.01%) to treat cell debris, and the mixture was incubated at 30 °C for 1 h with 100 rpm shaking (reciprocal). The treated cell debris was removed via centrifugation at 9100*g* for 2 min and re-suspended in 20 mM Tris–HCl (pH 6.8) as the insoluble fraction after TritonX-100 treatment. Each sample was analyzed using SDS-PAGE and PaE was detected through western blot analysis.

### PaE activity

Biodegradable plastic degradation activity was evaluated using emulsified PBSA (Bionolle EM-301; Showa Denko K. K., Tokyo, Japan), as described previously (Kitamoto et al. 2011). Briefly, a reaction mixture containing 20 mM Tris–HCl (pH 6.8), 0.045 (w/v)% emulsified PBSA and supernatant of the yeast culture or purified PaE was incubated at 30 °C for 15 min with 180 strokes/min shaking. After addition of the culture supernatant, the decrease in absorbance of the PBSA emulsion was measured at 660 nm (OD_660_). One unit (U) of PBSA degradation activity was defined as the activity that decrease OD_660_ by 1 in 1 mL reaction solution in 1 min. All analyses were performed in triplicate.

### Production and detection of MELs

Cells were cultivated in MEL production medium (1 g/L yeast extract, 3 g/L NaNO_3_, 0.3 g/L KH_2_PO_4_ and 0.3 g/L MgSO_4_·7H_2_O) containing 2 (w/v)% soybean oil at 30 °C for 4 days. The MELs produced were detected using thin-layer chromatography (TLC), as described previously (Morita et al. 2007). The method can be summarized as follows: cell culture was mixed with an equal volume of ethyl acetate, and 10 μL of this ethyl acetate extract was analyzed through TLC using chloroform: methanol: 12% NH_4_OH = 65: 15: 2 (v/v/v) as an eluent. MELs were detected by spraying with 2% anthrone-sulfate reagent and heating at 90 °C for 5 min. A purified mixture of MEL-A, MEL-B and MEL-C was used as a reference.

### The effects of surfactant addition on PaE production by strain ΔPa*EMT1*

ΔPa*EMT1* was cultivated in *m*-3×FMM containing 8 (w/v)% xylose supplemented with 0.01 (w/v)% of MELs, Tween20, BRIJ35, TritonX-100, SDS, sodium laurate (C12Na) or sodium stearate (C18Na). After 4 days of cultivation, the PaE activity in the culture supernatant was analyzed.

### Analysis of Pa*CLE1* expression levels through quantitative real-time PCR

The gene expression levels of Pa*CLE1*, encoding PaE, were analyzed through quantitative real-time PCR (qRT-PCR). *P. antarctica* GB-4(0) and strain ΔPa*EMT1* were cultivated in *m*-3×FMM medium containing 8 (w/v)% xylose with or without 0.01 (w/v)% TritonX-100. After 3 days of cultivation, cells were harvested from 30 mL of culture by centrifugation at 800*g* for 3 min and washed with distilled water twice. Total RNA was isolated from each sample with ISOGEN (Nippon Gene, Tokyo, Japan) according to the manufacturer’s instructions, and the total concentration of purified RNA was determined using a Nanodrop fluorometer (Nano Drop Technologies, Wilmington, DE, USA). Then, mRNA was purified with the Oligotex-dT30 < Super > mRNA Purification Kit (Takara Bio, Shiga, Japan). cDNA was synthesized from a template of 50 ng mRNA using the SuperScript VILO cDNA synthesis kit (Invitrogen) according to the manufacturer’s instructions. Primers for qRT-PCR were designed using Primer 3 (http://bioinfo.ut.ee/primer3-0.4.0/primer3/) (Table 1). The actin gene was used as a housekeeping gene. qRT-PCR was performed using Fast SYBR Green Master Mix (Applied Biosystems, Foster City, CA, USA) and visualized with LightCycler 2.0 with LightCycler software (ver. 5.0; Roche Diagnostics, Basel, Switzerland). Primer specificity was examined via melting curve analysis. Gene expression levels were quantified using the following equation: gene expression level = 2^−[Ct(Pa*CLE*1)−Ct(actin)]^. The accession numbers of the actin gene and Pa*CLE1* are LC466625 and LC276896, respectively.

## Results

### Deletion of Pa*EMT1* in strain GB-4(0)

**Fig. 1:**
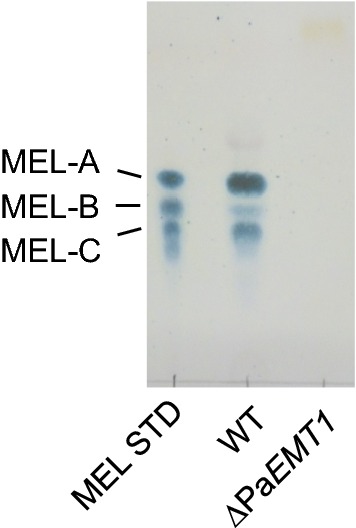
Pa*EMT1* deletion through homologous recombination in *P. antarctica* GB-4(0). Thin-layer chromatography (TLC) analysis of mannosylerythritol lipid (MEL) production by wild-type and Pa*EMT1*∆::NAT (∆Pa*EMT1*). MEL STD, MEL standard containing MEL-A, MEL-B and MEL-C

To obtain the MEL biosynthesis-deficient strain of GB-4(0), Pa*EMT1* was deleted and replaced with natMX4 through homologous recombination (Additional file 1: Figure S1A). The transformants generated on YPD plates with clonNAT were picked and analyzed using colony PCR to confirm Pa*EMT1* deletion (Additional file 1: Figure S1B). The inner region (1.0 kb) of *PaEMT1* was amplified via PCR with primers PaEMT1_inner_F1 (Primer A) and PaEMT1_inner_R1 (Primer B) from the wild-type colonies, but not from the transformants. On the other hand, amplification of the gene fragment Pa*EMT1*::natMX4 with primers PaEMT1_up_F2 (Primer C) and NATfragment_R1 (Primer D) was detected from the transformants. The transformants were defective in terms of MEL production when the cells were grown in MEL production medium, whereas the wild-type cells produced MELs (Fig. 1). These results indicate that Pa*EMT1* was replaced with natMX4, and that MEL biosynthesis was defective in the transformants, showing that Pa*EMT1* is essential for MEL biosynthesis in GB-4(0).

### PaE activity of strain ΔPa*EMT1*

**Fig. 2:**
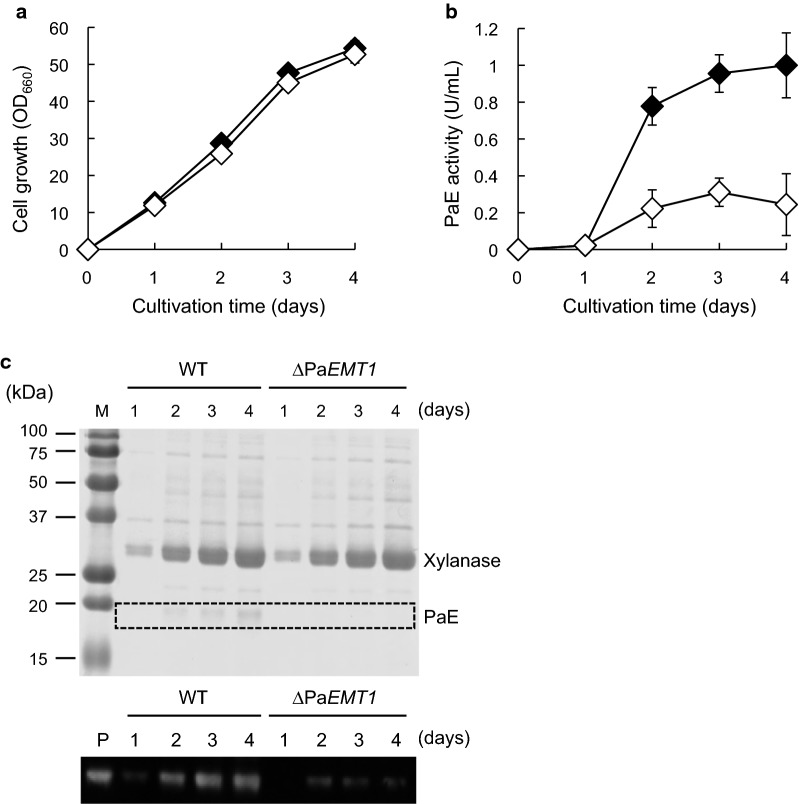
Time course of PaE production by strain ∆Pa*EMT1* in *m*-3×FMM (fungal minimum medium) supplemented with 8% xylose. **a** Cell growth of wild-type (closed diamonds) and ∆Pa*EMT1* (open diamonds). **b** Plastic-degrading enzyme (PaE) activity of wild-type (closed diamonds) and ∆Pa*EMT1* (open diamonds). **c** sodium dodecyl sulfate polyacrylamide gel electrophoresis (SDS-PAGE) and PaE detection through western blot analysis of culture supernatants of wild-type and ∆Pa*EMT1.* M, marker; P, purified PaE loaded as a standard. The amount of each culture supernatant loaded in the gel was 10 μL for Coomassie brilliant blue (CBB) staining and western blotting. The results of the cell growth and PaE activity assays are shown as the average of three different experiments. *Error bars* show standard deviations

To investigate the effect of Pa*EMT1* deletion on PaE production, strains GB-4(0) and Pa*EMT1*-deletion strain Pa*EMT1*∆::NAT (∆Pa*EMT1*) were cultivated in *m*-3×FMM-8% xylose. Cell growth of ΔPa*EMT1* was the same as that of the wild-type (Fig. 2a). Contrary to our expectation, PaE activity in the culture supernatant of ΔPa*EMT1* (0.2 ± 0.2 U/mL, 4 days cultivation) was clearly lower than that of the wild-type (1.0 ± 0.2 U/mL, 4 days cultivation) (Fig. 2b). In SDS-PAGE and western blot analyses, the signal corresponding to PaE from ΔPa*EMT1* was weaker than that from the wild-type (Fig. 2c). This result suggested that PaE expression and/or secretion decreased with defective MEL production. On the other hand, strain GB-4(0) secretes large amounts of 33-kDa endo-β-xylanase in the presence of xylose (Watanabe et al. 2015). We observed similar signal intensity corresponding to xylanase in the culture supernatants of ΔPa*EMT1* and wild-type cells (Fig. 2c). In addition, weak signals corresponding to other unknown proteins from ΔPa*EMT1* were the same as those from the wild-type. Based on these results, MEL biosynthesis is likely to contribute strongly to PaE production.

### Complementation of Pa*EMT1* deletion

**Fig. 3:**
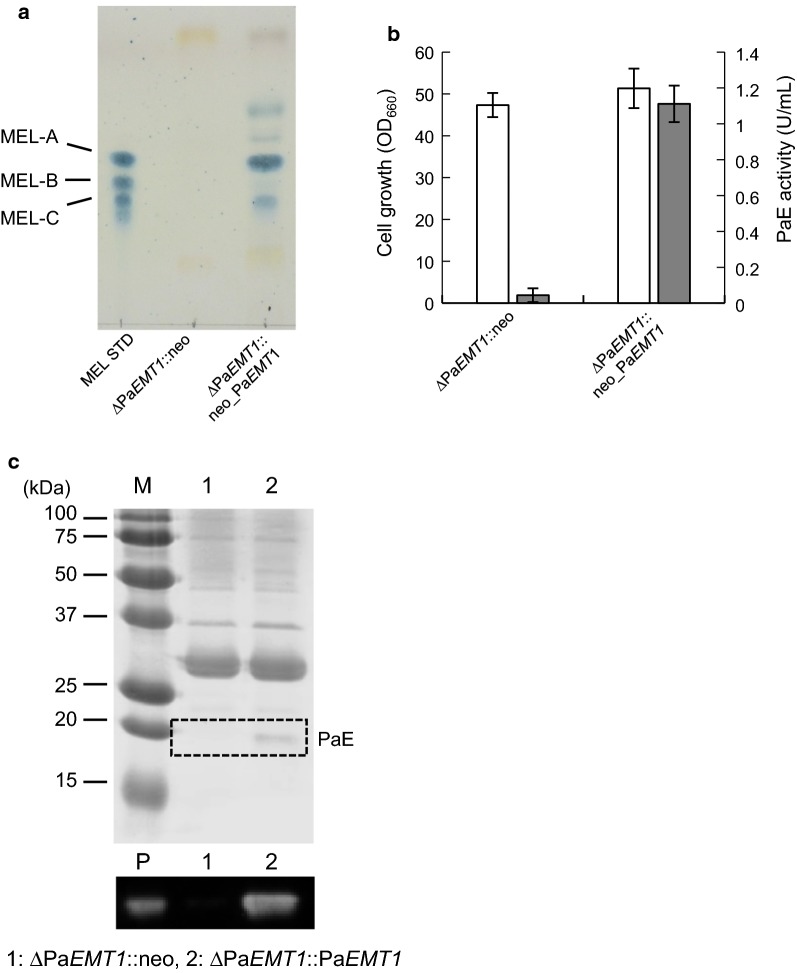
PaE production by strain ∆Pa*EMT1* harboring Pa*EMT1* in 3×FMM medium supplemented with 8% xylose. **a** TLC analysis of MEL production by the Pa*EMT1*-complemented strain. ∆Pa*EMT1*::neo: ∆Pa*EMT1* strain harboring pUXV1_neo, ∆P*aEMT1*::neo_Pa*EMT1*: ∆Pa*EMT1* strain harboring pUXV1_neo-PaEMT1. **b** Cell growth (white) and PaE activity (gray). **c** SDS-PAGE and PaE detection through western blot analysis of culture supernatant. M, marker; P, purified PaE; Lane 1, ∆PaEMT1::neo; Lane 2, ∆Pa*EMT1*::Pa*EMT1*. The amount of each culture supernatant loaded in the gel was 10 μL for CBB staining and western blotting. The results of the cell growth and PaE activity assays are shown as the average of three different experiments. *Error bars* show standard deviations

To confirm the phenotypic changes due to loss of MEL production described above, Pa*EMT1* deletion was complemented through introduction of Pa*EMT1* with the plasmid pUXV1_neo-PaEMT1, and pUXV1_neo was used as a control. The recombinant strains ΔPa*EMT1* harboring pUXV1_neo-PaEMT1 or pUXV1_neo were cultivated in MEL production medium containing 2 (w/v)% soybean oil and 200 μg/mL G418 for 4 days at 30 °C. In TLC analysis, MEL production was recovered in ΔPa*EMT1* harboring pUXV1_neo-PaEMT1 (Fig. 3a). Cell growth of both strains was identical (Fig. 3b). PaE activity and secretion in the culture supernatant were recovered in ΔPa*EMT1* harboring pUXV1_neo-PaEMT1 (Fig. 3b, c) in addition to MEL production. These results strongly support MEL biosynthesis being necessary for PaE secretion or production in strain GB-4(0).

### The effects of surfactant addition on PaE production in strain ΔPa*EMT1*

**Fig. 4:**
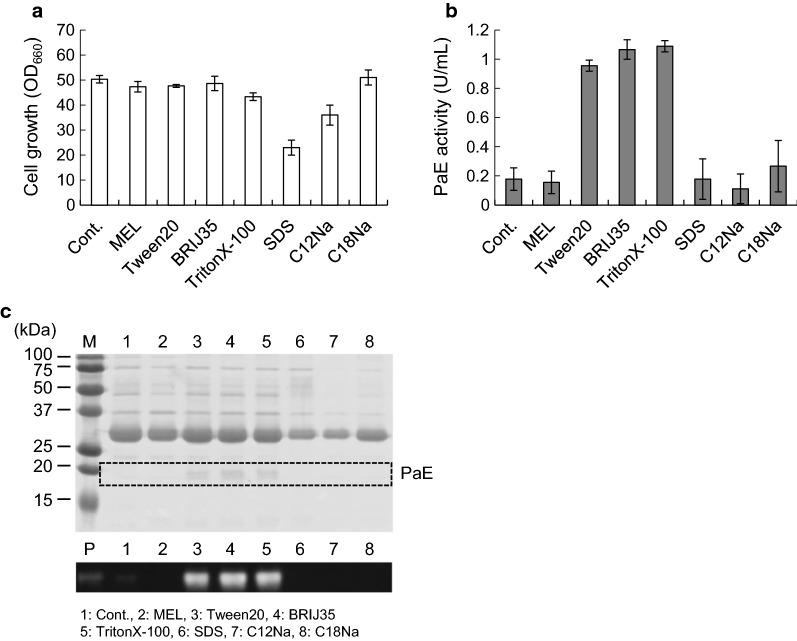
PaE production by strain ∆Pa*EMT1* in *m*-3×FMM medium supplemented with 8% xylose and 0.01% surfactants. **a** Cell growth. **b** PaE activity. **c** SDS-PAGE and PaE western blot analysis of culture supernatant. M, marker; P, purified PaE; Lane 1, control; Lane 2, MEL; Lane 3, Tween20; Lane 4, BRIJ35; Lane 5, TritonX-100; Lane 6, SDS; Lane 7, sodium dodecanoate (C12Na); Lane 8, sodium octadecenoate (C18Na). The amount of each culture supernatant loaded in the gel was 10 μL for CBB staining and western blotting. The results of the cell growth and PaE activity assays are shown as the average of three different experiments. *Error bars* show standard deviations

We then investigated the effects of 0.01% MELs and various synthetic surfactants on PaE production in ΔPa*EMT1*. While SDS and C12Na affected cell growth, the other additions (MELs, Tween20, BRIJ35, TritonX-100, C18Na) generally maintained normal growth of ∆Pa*EMT1* (Fig. 4a). The PaE activities in the cultures supplemented with Tween20, BRIJ35 and TritonX-100 were markedly elevated compared to the control (Fig. 4b). A signal corresponding to PaE was detected in all cultures supplemented with Tween20, BRIJ35 and TritonX-100 in SDS-PAGE and western blotting analyses (Fig. 4c). However, PaE activity and the PaE signal did not recover with 0.01% MEL addition, even though MEL is the native surfactant of strain GB-4(0).

**Fig. 5:**
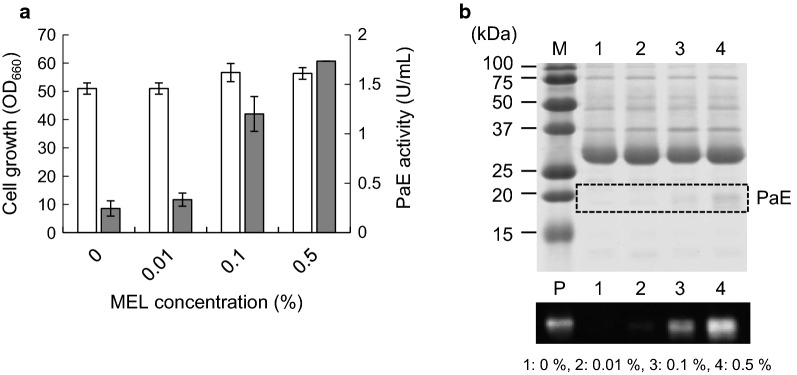
PaE production by strain ∆Pa*EMT1* in *m*-3×FMM medium supplemented with 8% xylose and various concentrations of MEL. **a** Cell growth (white) and PaE activity (gray). **b** SDS-PAGE and western blot analysis of culture supernatant. M, marker; P, purified PaE. The amount of each culture supernatant loaded in the gel was 10 μL for CBB staining and western blotting. The results of the cell growth and PaE activity assays are shown as the average of three different experiments. *Error bars* show standard deviations

The effect of varying the concentration of MELs was tested. A higher concentration of MELs did not affect cell growth (Fig. 5a). Recovery of PaE secretion was observed in the presence of 0.1 and 0.5% MELs based on PaE activity and PaE detection through western blotting (Fig. 5a, b). Based on this result, a higher concentration of MEL was required to recover PaE productivity compared to other surfactants.

### Relative expression level of Pa*CLE1* in ∆Pa*EMT1* strain

Although the protein secretion/degradation pathway is unknown in *P. antarctica*, we analyzed the expression of Pa*CLE1*, encoding PaE, through qRT-PCR to determine whether PaE production deficiency depends on transcriptional regulation or an unknown protein degradation pathway. The expression level of the gene was the same in ∆Pa*EMT1* (2.26 ± 0.22), wild-type (2.12 ± 0.25) and ∆Pa*EMT1* grown in the medium supplemented with 0.01% TritonX-100 (2.10 ± 0.51). This result indicates that Pa*CLE1* is transcribed normally in ∆Pa*EMT1*, but that the gene product secretion system was defective.

### Detection of PaE in cell-free extract

**Fig. 6:**
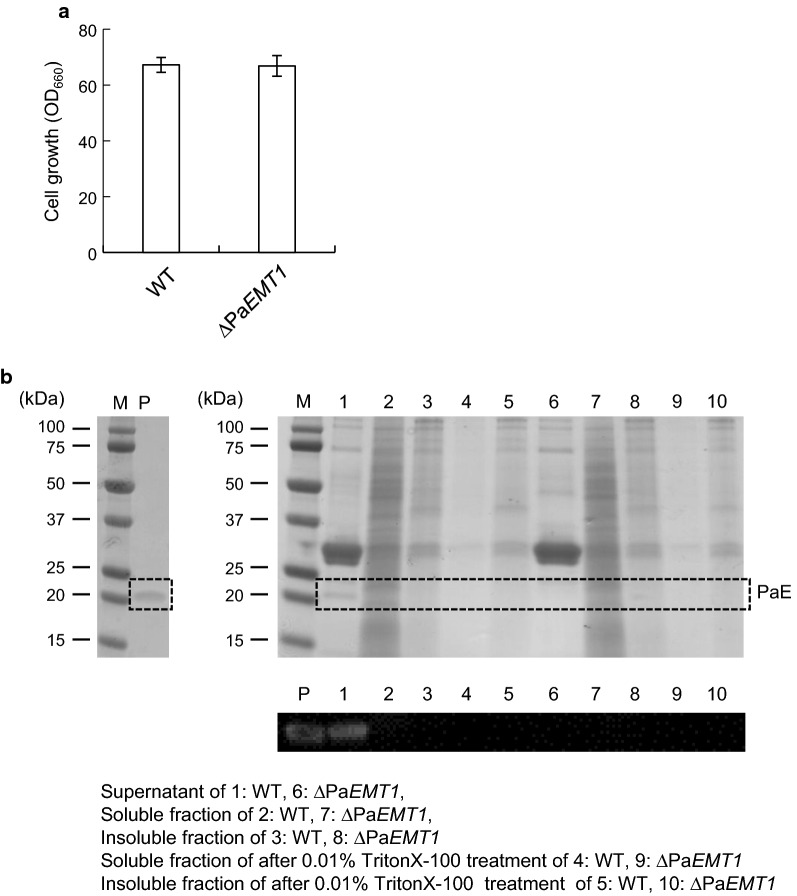
SDS-PAGE and PaE western blot analysis of culture supernatant and cell-free extract of GB-4(0) and strain ∆Pa*EMT1*. **a** Cell growth. **b** SDS-PAGE and PaE detection through western blot analysis. M, marker; P, purified PaE; Lane 1, supernatant of wild-type; Lane 2, soluble fraction of wild-type; Lane 3, insoluble fraction of wild-type; Lane 4, soluble fraction after 0.01% TritonX-100 treatment of wild-type; Lane 5, insoluble fraction after 0.01% TritonX-100 treatment of wild-type; Lane 6, supernatant of ∆Pa*EMT1*; Lane 7, soluble fraction of ∆Pa*EMT1*; Lane 8, insoluble fraction of ∆Pa*EMT1*; Lane 9, soluble fraction after 0.01% TritonX-100 treatment of ∆Pa*EMT1*; Lane 10, insoluble fraction after 0.01% TritonX-100 treatment of ∆Pa*EMT1*. The amount of each sample loaded in the gel was 20 μL for CBB staining and western blotting. *Error bars* show standard deviations

As mentioned above, PaE was not secreted into the culture supernatant of ∆Pa*EMT1*, although the Pa*CLE1* gene was expressed at the same level as in the wild-type. From these results, we speculated that PaE secretion failed due to the loss of MEL production. To test this hypothesis, cell-free extract was prepared and analysis of intracellular PaE was carried out using SDS-PAGE and western blotting. The detailed preparation method for cell-free extract is summarized in Additional file 1: Figure S2. According to Fig. 6, PaE was detected only in the culture supernatant of strain GB-4(0) (Fig. 6b, Lane 1), and showed no signals in the soluble or insoluble fractions of either strain GB-4(0) (Fig. 6b, Lane 2 and 3) or ∆Pa*EMT1* (Fig. 6b, Lane 7 and 8). Additionally, precipitate of the insoluble fraction was washed using 0.01% TritonX-100 for 1 h at 30 °C (Additional file 1: Figure S2). Because PaE productivity recovered with the addition of 0.01% TritonX-100 (Fig. 4), we expected that PaE would be eluted after 0.01% TritonX-100 treatment if PaE was insolubilized. However, no signal was present in the fraction obtained after TritonX-100 treatment (Fig. 6b, Lane 9 and 10). These results suggest that the gene Pa*CLE1* was not translated to PaE or that expressed PaE was degraded immediately in ∆Pa*EMT1*.

## Discussion

In this study, we found that the biosynthesis of extracellular glycolipids known as MELs contributes to production of an extracellular esterase, PaE, in *P. antarctica* GB-4(0). Furthermore, we demonstrated that addition of various surfactants including MEL complement this deficiency; thus, MEL biosynthesis is required for PaE production in strain GB-4(0).

A gene cluster consisting of five genes responsible for MEL biosynthesis has been reported in *U. maydis* UM521 and *P. antarctica* T-34, *P. antarctica* JCM10317^T^, *P. aphidis* DSM70725 and *P. tsukubaensis* NBRC1940 (Hewald et al. 2006; Lorenz et al. 2014; Morita et al. 2013a; Saika et al. 2014, 2016). Of these genes, a gene encoding glycosyltransferase (corresponding to Emt1 of *U. maydis* and PaEmt1p of *P. antarctica*) is essential for MEL production in *U. maydis* UM521 and *P. antarctica* T-34, as reported previously (Hewald et al. 2005; Morita et al. 2010). Pa*EMT1* from strain GB-4(0) shares high amino acid sequence identity with *U. maydis* UM521, *P. antarctica* T-34 and *P. antarctica* JCM10317; 75, 100 and 95%, respectively. The Pa*EMT1*-deletion strain exhibits MEL biosynthesis deficiency, and the phenotype was complemented by transformation with Pa*EMT1.* Thus, Pa*EMT1* is essential for MEL production in GB-4(0), in accordance with results from strain T-34 (Morita et al. 2010).

PaE activity recovered with the addition of synthesized 0.01% Tween20, BRIJ35, or TritonX-100, while the other surfactants, including 0.01% MEL, did not lead to such activity (Fig. 4). The critical micelle concentration (CMC) of each surfactant was as follows: MEL: 0.0027 mM (1.8 × 10^−4^%), Tween20: 0.06 mM (0.0074%), BRIJ35: 0.09 mM (0.011%), TritonX-100: 0.24 mM (0.015%), SDS: 8 mM (0.23%), C12Na: 27.5 mM (0.61%), and C18Na: 0.5 mM (0.015%). A concentration of 0.01% is lower than the CMC for BRIJ35, TrironX-100, SDS, C12Na and C18Na; therefore, CMC is likely unrelated to the recovery of PaE production. In addition, we estimated the effect of the initial content of TritonX-100 on PaE production with addition of 0, 0.2, 0.5, 1, and 5 CMC. As shown in Additional file 1: Figure S3, recovery of PaE production was observed at 0.2–5 CMC, supporting the hypothesis described above. SDS, C12Na and C18Na, which did not have an effect on recovery of PaE, are categorized as anionic surfactants, while MEL, Tween20, BRIJ35 and TritonX-100 are categorized as non-ionic surfactant. From this result, the type of surfactant might affect the ability to recover PaE production. According to Fig. 5, a higher concentration of MEL was needed to recover PaE production. *P. antarctica* is known to produce large amounts of MEL (over 14% in culture medium) (Kitamoto et al. 2001), which could be related to the requirement for a high concentration of MEL, but further investigation is needed to understand the mechanism of PaE secretion.

Fukuoka et al. (2016) showed that PaE activity decreased with the addition of MELs at final concentrations of 5 and 50 mg/L (0.0005 and 0.005%, respectively). While the MEL concentrations shown in Fig. 5 were 1000 times higher than those in previous reports, PaE activities were not inhibited. The culture supernatant used for the PaE activity assay shown in Fig. 5 was analyzed through TLC (Additional file 1: Figure S4). According to the results, the added MEL was not detected in the supernatant of every culture (Additional file 1: Figure S4A). On the other hand, added MEL was detected in the precipitate that included cells, but the amount of MEL was lower than the MEL standard (Additional file 1: Figure S4B). Based on these results, addition of MEL did not inhibit PaE activity because the MEL was precipitated during centrifugation, and some of the added MEL may have degraded during cultivation.

As the Pa*CLE1* expression level of strain ∆Pa*EMT1* was the same as that of the parent strain, recovery of deficient PaE production with surfactants may be linked to post-translational events, such as protein trafficking and degradation pathways. Because these amphiphilic compounds will interact with the cell membrane, various intracellular pathways related to cell integrity, e.g., biosynthesis of components of the cell membrane and cell wall and the protein kinase C pathway, respond to treatment with detergents. Moreover, the absence of MELs may induce deficiency of protein trafficking in transport vesicles, because the composition of intracellular membranes affects their function (Phillips et al. 2009; Alexander et al. 2011; Iwamoto and Oiki 2013). After incorporation into the intracellular membrane via endocytosis, the amphiphilic compounds may complement the absence of MELs in the intracellular membrane, although the function of MELs in protein sorting is unknown. Further study of the molecular cell biology of *P. antarctica*, including protein trafficking, degradation, secretion, and the localization and transportation pathways of MELs, will clarify the mechanisms of PaE production.

## Additional file

**Additional file 1: Figure S1.** PCR analysis of the parental strain GB-4(0) and strain ΔPa*EMT1*. The primer sets used in PCR amplification to assess Pa*EMT1* deletion (A). Agarose gel electrophoresis of amplified DNA fragments to confirm gene disruption. The gene fragments were amplified using PaEMT1_inner_F1 (Primer A) and PaEMT1_inner_R1 (Primer B), or PaEMT1_up_F2 (Primer C) and NATfragment_R1 (Primer D) (B). **Figure S2.** Schematic diagram of the procedure used to obtain cell-free extract. **Figure S3.** PaE production by strain ΔPa*EMT1* in *m*-3×FMM medium supplemented with 8 % xylose and various concentrations of TritonX-100. Cell growth (white) and PaE activity (gray) (A) and SDS-PAGE and western blot analyses of culture supernatant (B). CMC, critical micelle concentration; M, marker; P, purified PaE. The amount of each culture supernatant loaded in the gel was 10 μl for CBB staining and western blotting. The results of the cell growth and PaE activity assays are shown as the average of three different experiments. *Error bars* show standard deviations. **Figure S4.** TLC analysis of culture supernatants used for the PaE activity assay. Culture supernatants (A) and precipitates including cells (B).

## Data Availability

All data generated during this study are included in this article and its additional files.
