# Supplementary material for: Deficiency of biodegradable plastic-degrading enzyme production in a gene-deletion mutant of phyllosphere yeast, Pseudozyma antarctica defective in mannosylerythritol lipid biosynthesis
Source: AMB Express. 2019 Jul 6;9:100. doi: 10.1186/s13568-019-0825-2 (PMC6612326; doi:10.1186/s13568-019-0825-2)
Supplement: Supplementary file 1 — Additional file 1: Figure S1. PCR analysis of the parental strain GB-4(0) and strain ΔPaEMT1. The primer sets used in PCR amplification to assess PaEMT1 deletion (A). Agarose gel electrophoresis of amplified DNA fragments to confirm gene disruption. The gene fragments were amplified using PaEMT1_inner_F1 (Primer A) and PaEMT1_inner_R1 (Primer B), or PaEMT1_up_F2 (Primer C) and NATfragment_R1 (Primer D) (B). Figure S2. Schematic diagram of the procedure used to obtain cell-free extract. Figure S3. PaE production by strain ΔPaEMT1 in m-3×FMM medium supplemented with 8 % xylose and various concentrations of TritonX-100. Cell growth (white) and PaE activity (gray) (A) and SDS-PAGE and western blot analyses of culture supernatant (B). CMC, critical micelle concentration; M, marker; P, purified PaE. The amount of each culture supernatant loaded in the gel was 10 μl for CBB staining and western blotting. The results of the cell growth and PaE activity assays are shown as the average of three different experiments. Error bars show standard deviations. Figure S4. TLC analysis of culture supernatants used for the PaE activity assay. Culture supernatants (A) and precipitates including cells (B). [file 13568_2019_825_MOESM1_ESM.docx]

**Journal: AMB Express**

**Supplementary materials**

Deficiency of biodegradable plastic-degrading enzyme production in a gene-deletion mutant of phyllosphere yeast, *Pseudozyma antarctica* defective in mannosylerythritol lipid biosynthesis

Azusa Saika^1^, Hideaki Koike^2^, Tohru Yarimizu^3^, Takashi Watanabe^3,†^, Hiroko Kitamoto^3^, Tomotake Morita^1, *^

Research Institute for Sustainable Chemistry, National Institute of Advanced Industrial Science and Technology (AIST), Tsukuba Central 5-2, 1-1-1 Higashi, Tsukuba, Ibaraki 305-8565, Japan^1^, Bioproduction Research Institute, National Institute of Advanced Industrial Science and Technology (AIST), Tsukuba Central 6-9, 1-1-1 Higashi, Tsukuba, Ibaraki 305-8566, Japan^2^, Institute for Agro-Environmental Sciences, National Agricultural Food Research Organization (NARO), 3-1-3 Kannondai, Tsukuba, Ibaraki 305-8604, Japan^3^

^†^Present address: Gunma Industrial Technology Center, 884-1 Kamesato, Mae-bashi, Gunma 379-2147, Japan.

*Corresponding author

E-mail: [morita-tomotake@aist.go.jp](mailto:morita-tomotake@aist.go.jp)

Phone: +81-29-861-4426

Fax: +81-29-861-4457

**Figure S1:**

**Figure S1.** PCR analysis of the parental strain GB-4(0) and strain ΔPa*EMT1*. The primer sets used in PCR amplification to assess Pa*EMT1* deletion (A). Agarose gel electrophoresis of amplified DNA fragments to confirm gene disruption. The gene fragments were amplified using PaEMT1_inner_F1 (Primer A) and PaEMT1_inner_R1 (Primer B), or PaEMT1_up_F2 (Primer C) and NATfragment_R1 (Primer D) (B).

**Figure S2:**

**Figure S2.** Schematic diagram of the procedure used to obtain cell-free extract.

**Figure S3:**

**Figure S3.** PaE production by strain ΔPa*EMT1* in *m*-3×FMM medium supplemented with 8 % xylose and various concentrations of TritonX-100. Cell growth (white) and PaE activity (gray) (A) and SDS-PAGE and western blot analyses of culture supernatant (B). CMC, critical micelle concentration; M, marker; P, purified PaE. The amount of each culture supernatant loaded in the gel was 10 μl for CBB staining and western blotting. The results of the cell growth and PaE activity assays are shown as the average of three different experiments. *Error bars* show standard deviations.

**Figure S4:**

**Figure S4.** TLC analysis of culture supernatants used for the PaE activity assay. Culture supernatants (A) and precipitates including cells (B).
